# Patient's Perspective on the Impact of COVID-19 on Cancer Treatment in Nigeria

**DOI:** 10.1200/GO.21.00244

**Published:** 2022-02-14

**Authors:** Adedayo Joseph, Bankole Olatosi, Mohammad Rifat Haider, Bolanle C. Adegboyega, Nwamaka N. Lasebikan, Usman M. Aliyu, Musa Ali-Gombe, Mutiu A. Jimoh, Olusegun Abayomi Biyi-Olutunde, Opeyemi Awofeso, Omolara Aminat Fatiregun, Evaristus Oseiwe Oboh, Emmanuella Nwachukwu, Ismail H. Zubairu, Samuel A. Otene, Oluwatoyin I. Iyare, Temitope Andero, Alabi Babatunde Musbau, Azeezat Ajose, Adedayo A. Onitilo

**Affiliations:** ^1^ NSIA-LUTH Cancer Center, Lagos University Teaching Hospital, Lagos, Nigeria; ^2^ Health Services, Policy and Management, Arnold School of Public Health, University of South Carolina, Columbia, SC; ^3^ Department of Health Policy and Management, College of Public Health, University of Georgia, Athens, GA; ^4^ University of Nigeria Teaching Hospital, Enugu, Nigeria; ^5^ Usman Danfodiyo University Teaching Hospital, Sokoto, Nigeria; ^6^ Federal Teaching Hospital, Gombe, Nigeria; ^7^ University College Hospital, Ibadan, Oyo, Nigeria; ^8^ Lakeshore Cancer Center, Lagos, Nigeria; ^9^ University of Port-Harcourt Teaching Hospital, Port-Harcourt, Rivers State, Nigeria; ^10^ Lagos University Teaching Hospital, Idiaraba, Lagos, Nigeria; ^11^ Department of Radiology, Lagos State University Teaching Hospital (LASUTH), Lagos, Nigeria; ^12^ University of Benin Teaching Hospital, Edo, Nigeria; ^13^ National Hospital, Abuja, Federal Capital Territory, Nigeria; ^14^ Ahmadu Bello University Teaching Hospital, Kaduna, Nigeria; ^15^ Federal Medical Centre, Makurdi, Borno, Nigeria; ^16^ Alex Ekwueme Federal Teaching Hospital Abakaliki, Ebonyi, Nigeria; ^17^ Eko Hospital, Lagos, Nigeria; ^18^ Federal Medical Centre, Abeokuta, Ogun, Nigeria; ^19^ Department of Oncology, Marshfield Clinic Health System, Marshfield, WI

## Abstract

Because of the global COVID-19 pandemic, health care organizations introduced guidelines for modifications to health and cancer medical care delivery to mitigate transmission and ensure quality health outcomes. To examine the extent and impact of these modifications on oncology service disruptions in Nigeria, we surveyed oncology patients across selected public and private cancer treatment centers.

**MATERIALS AND METHODS:**

Participating in the study were 15 tertiary cancer treatment centers across 12 Nigerian states. We recruited adult patients with cancer (18+ years) on active treatment to complete a self-administered survey on cancer care during COVID-19. We conducted descriptive and multivariate data analysis using Stata 16.1.

**RESULTS:**

Respondents were (n = 1,072), female (65.7%), ages 18-49 years (50.3%), and married (80.7%). The top two cancers were breast and prostate. Overall, 17.3% of respondents reported disruptions to cancer care, and more than half (51.0%) reported difficulties accessing care. Changes in chemotherapy regimens or route of administration were reported in 8.4% of respondents. Odds for any disruption were highest for older patients, western states, patients with prostate cancer, and patients with two or more flu symptoms. Odds for radiotherapy cancellation were highest for older patients, those with prostate cancer, and those with medium service perception.

**CONCLUSION:**

This study investigated COVID-19–influenced cancer treatment disruptions in Nigeria. Patients with cancer experienced significant disruptions to cancer care. Vulnerable patients are most likely to be negatively affected. Policies and strategies aimed at minimizing service disruptions while maintaining cancer patients' safety should be a priority for all health care institutions in the COVID-19 era.

## BACKGROUND

In 2019, a cluster of patients with a pneumonia-like illness was reported to the WHO Country Office in Wuhan, a city in Central China. This signaled the beginning of the global COVID-19 pandemic.^1^ This pneumonia was linked to a causative novel strain of coronavirus, initially named novel coronavirus (2019-nCov) and later renamed SARS-CoV-2, and the disease was termed COVID-19. On January 30, 2020, the WHO declared the current novel COVID-2019 epidemic a public health emergency of international concern.^2^

**CONTEXT**

**Key Objective**
The COVID-19 pandemic created a global health care crisis on an unprecedented scale. Maintaining access to care in the face of social distancing measures, lockdowns, and health care shortages was a challenge. Patients with cancer experienced service interruptions as attention turned almost exclusively to mitigating the impact of the virus.
**Knowledge Generated**
Fifteen of the largest cancer treatment centers across Nigeria were assessed for service interruptions experienced by patients with cancer. Seventeen percent of patients with cancer reported experiencing interruptions to their treatment. More than half (51.0%) of the patients reported difficulties in accessing their care as a result of changes to treatment regimens, postponement, or cancellation of treatments. Older age groups, people diagnosed with prostate cancer, and people with two or more possible symptoms of COVID experienced the greatest interruptions.
**Relevance**
Policies and strategies to ensure maintenance of uninterrupted care without compromising patient safety should be put in place as standard of practice in cancer centers.

The pandemic heralded an unprecedented period of disruption in global health and economic activities.^3^ Since its emergence, COVID-19 has presented a significant challenge to health care access globally, pushing treatment to the margins or on hold for many chronic conditions including cancer. The mitigation measures put in place across the world to halt the spread of the virus also resulted in diverted attention and care for chronically ill patients.^4^ Many of the protective measures resulted in delays and disruptions to health care services, possibly posing a risk to survival for the patients with cancer.^5^

Several health care and cancer treatment facilities and governing bodies created guidelines for the care of patients during this period for the protection of both the patient and the physician.^6^ Treatments and clinical visits were canceled, postponed, adjusted, or modified to reduce exposure to patients and physicians.^7^ To curb the spread of the virus, the Nigerian Government, like many governments, put in place lockdowns in major states and cities. Major metropolitan areas across states, such as Lagos, Ogun, Rivers, Kano, and the Federal Capital Territory in Abuja, went into full lockdown, invariably creating difficulties with access to care for patients. By December 2020, Nigeria had recorded more than 97,000 cases of COVID-19 with 1,500 deaths, for a mortality rate of 1.49%.^8^ A total of 42,161 cases of COVID-19 were recorded in the 12 states covered in this study or approximately 68.5% of the reported total caseload for Nigeria.^8^

This project assessed patient-reported impact of the COVID-19 pandemic on access to cancer care across the three major geopolitical zones in Nigeria: North, South, and West. We assessed patient-reported service disruptions and the patients' perception of the impact of these changes on their cancer care. We also investigated patient knowledge of COVID-19 transmission and presentation and their perceptions of the virus and disease in relation to cancer care.

## MATERIALS AND METHODS

### Study Area

Nigeria is the most populous country in Africa with an estimated population of 200 million people, reporting an estimated 124,815 new cancer cases and 78,889 cancer-related deaths in 2020 alone.^9^ There are 54 health care centers in Nigeria providing tertiary-level health care services, with nine of them being classified as comprehensive cancer centers. Of these, eight centers had radiotherapy machines at the time of this study.^10^ Participating in this study were 15 centers located in 12 states across all six geopolitical zones in Nigeria. All participating centers offer tertiary-level expert cancer care. Of the centers, 12 are government-owned public hospitals offering tertiary-level health care services, of which nine are academic health centers, two centers are private cancer centers, and one center is a public-private partnership structured institution attached to one of the oldest and foremost government-owned academic health centers in the country. The participating centers were grouped into North, South, and West on the basis of geopolitical divides.

The nine government-owned participating academic health centers include the National Hospital Abuja (North); Ahmadu Bello University Teaching Hospital, Zaria (North); Usman Danfodiyo University Teaching Hospital, Sokoto (North); NSIA-LUTH Cancer Centre, Lagos (South); University College Hospital, Ibadan Oyo State (South); Lagos State University Teaching Hospital (South); University of Nigeria Teaching Hospital, Enugu (South); University of Port-Harcourt Teaching Hospital, Rivers State (South); and University of Benin Teaching Hospital, Benin (South). Four centers are government-owned, nonacademic health centers: Federal Medical Center Abeokuta, Ogun state (South West); Federal Teaching Hospital Abakaliki, Ebonyi state (South East); Federal Teaching, Gombe state (North East); and Federal Medical Center, Makurdi (North Central). Two health centers are privately owned centers offering expert cancer services: Lakeshore Cancer Center, Lagos (South West) and Eko Hospital, Lagos (South West).

### Study Design

This multicenter cross-sectional survey-based study was carried out in 15 centers across three major geopolitical zones in Nigeria: North, South, and West. One center was randomly selected from each zone. We obtained institutional review board approval for the study from the National Health Research and Ethics Committee of Nigeria.

All adult patients (≥ 18 years) with histologically diagnosed cancer and on active treatment being seen at participating centers during the study period (April 2020 to July 2020) were approached by a study investigator for each site. Patients who had not commenced treatment for cancer or who had completed their full course of cancer treatment were excluded.

We provided a uniform self-administered paper-based survey questionnaire to consenting patients on clinical days at all participating centers during the study period. The survey tool was designed by the research team and assessed self-reported presence of possible COVID-19 symptoms, knowledge of modes of transmission and signs or symptoms of COVID-19, use of protective personal equipment by medical staff, protective measures instituted by respective centers, and disruptions of cancer care services. The survey assessed patient's perception of cancer-specific treatment disruptions as its primary outcome. We designed and adapted survey questions from the literature to the Nigerian context.^11-14^ The survey tool elicited information regarding the sociodemographic profile, location, and clinical history (including cancer type and treatment given) of study participants. Investigators for all 15 centers reviewed the instrument, which was pretested on 30 participants with cancer at the NSIA-LUTH Cancer Centre in Lagos. We modified the tool on the basis of the results of the pretest.^11-14^

### Service Disruptions

We explored three different service interruptions as reported by patients on treatment for cancer as the primary outcome: disruptions to radiotherapy, disruptions to chemotherapy, and change in chemotherapy administration route from injection to oral using the uniform self-administered patient survey tool. We also assessed any occurrence of any kind of service disruption at all as a composite outcome (defined as the presence of at least one service disruption). Assessed variables included difficulties experienced accessing care and satisfaction with care received during the pandemic. Perception about the impact of COVID-19 on related service access was measured by asking questions that assessed whether there was adequate personal protective equipments, whether it was difficult to access cancer care because of lockdown measures, whether it was difficult to reach/access their doctors, and whether it was difficult to get a treatment prescription. If participants responded yes, they were assigned a score of 1, and if the response was no, they were assigned a score of 0. If the total score was 0-1, the perceived adverse impact of COVID-19 on cancer treatment service provision was regarded as low, 2 as medium, and 3-4 was regarded as high.

### Independent Variables

We examined explanatory variables including patient sociodemographic characteristics such as age (18-49, ≥ 50 years), sex (female or male), marital status (single, married, or divorced/widowed), ethnicity (Hausa, Igbo, Yoruba, or others), religion (Christianity, Islam, Atheism, or others), level of education attained (no education, primary, secondary, or tertiary), and monthly household income (< $100 US dollars [USD], $100-$500 USD, or > $500 USD per month). Clinical data, including the type of cancer and any comorbidity, were also included.

### Statistical Analysis

Descriptive statistics using univariate analysis, bivariate analysis using Fisher's exact test, and multivariable analyses using multiple logistic regression were performed to examine the association between service disruptions and COVID-19–related factors among patients with cancer in Nigeria. We used Fisher's exact test for bivariate analysis because some categories of variables had sparse data. We used penalized (Firth's) logistic regression models for multivariable analysis to account for small-sample bias with our sparse data problem.^15-17^ Sociodemographic variables included in the multivariable model were age, sex, religion, ethnicity, marital status, regions of residence, education, cancer type, and comorbidities. All analyses were performed using Stata 16.1 (StataCorp, 2019).^18^

## RESULTS

A total of 1,337 patients with cancer attending oncology clinics at the study centers were approached for the study, of which 1,179 patients consented to participate in the study, and a final of 1,072 surveys were completely filled and used for the study, resulting in a response rate of 80.7%.

### Sociodemographic Characteristics of Patients With Cancer

Table 1 presents the sociodemographic characteristics of patients with cancer who consented to survey participation. A majority of participants were 18-49 years (50.3%), female (65.7%), Christian (77.5%), married (78.5%), had a monthly household income of $100-$500 USD (58.2%), and reported no comorbidities (69.8%). Of the participants, two fifths were of Igbo ethnicity (40.8%), and most had secondary education or higher. Survey participants were distributed equally across the three major geopolitical regions of Nigeria.

**TABLE 1:**
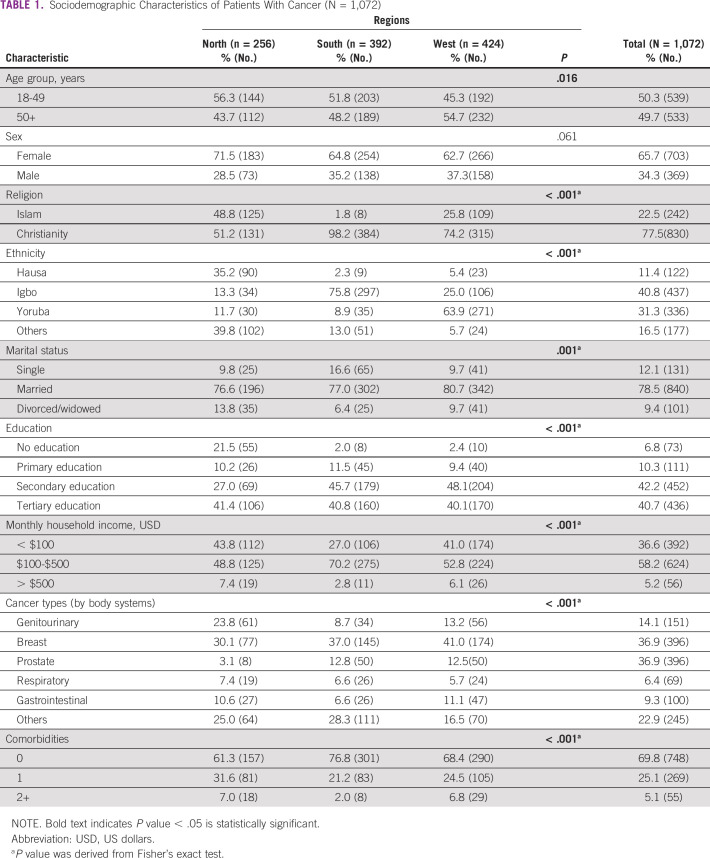
Sociodemographic Characteristics of Patients With Cancer (N = 1,072)

Table 1 also highlights regional differences for patients with cancer. Age differences existed by region, with Northern and Southern patients younger than those from the West. Most female patients had breast cancer, were Christian, were married, and had a reported monthly household income of $100-$500 USD across all regions. Ethnicity for the South was mostly Igbo (75.8%), whereas majority of patients in the West were Yoruba (63.9%). Overall, the South and West had higher levels of education compared with the North. Majority of respondents from the South reported the highest annual household incomes. More than half of the respondents reported having none of the listed possible symptoms of COVID-19 at the time of the study; 27% reported having one symptom, and 8.1% reported two or more symptoms.

### Service Disruption and Cancellation Experienced by Patients With Cancer

**FIG 1:**
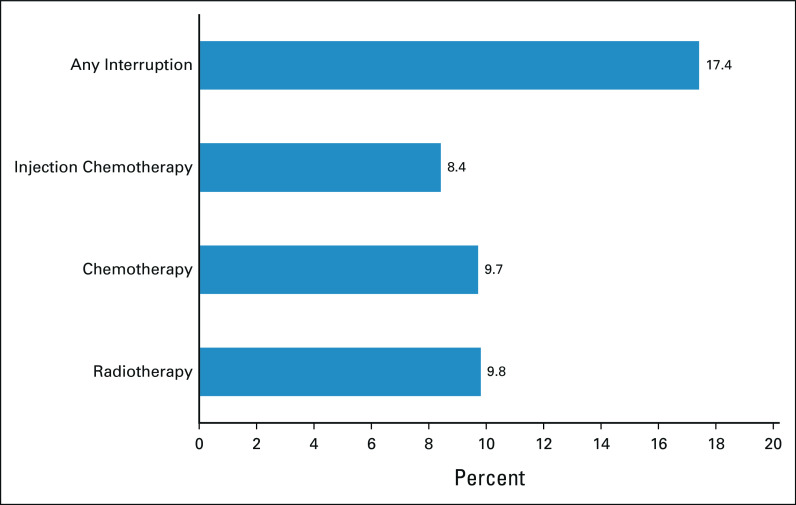
Service disruption experienced by patients with cancer.

Almost one in five patients (17.4%) reported any disruptions in cancer care because of COVID-19 mitigation measures (Fig 1). Cancellations occurred in radiotherapy (9.8%) and chemotherapy (9.7%); < 10% of respondents reported changing chemotherapy from injection to an oral route of administration.

Table 2 summarizes the results from the bivariate analyses of associations between different covariates and service disruptions because of COVID-19 for treatment types. Any disruption in cancer care services because of COVID-19 occurred more often among respondents age ≥ 50 years (*P* = .001), of the Islamic faith (*P* = .001), Yoruba ethnicity (*P* < .001), residents of Western region (*P* = .027), secondary education (*P* = .039), and a monthly household income < $100 USD (*P* < .001).

**TABLE 2:**
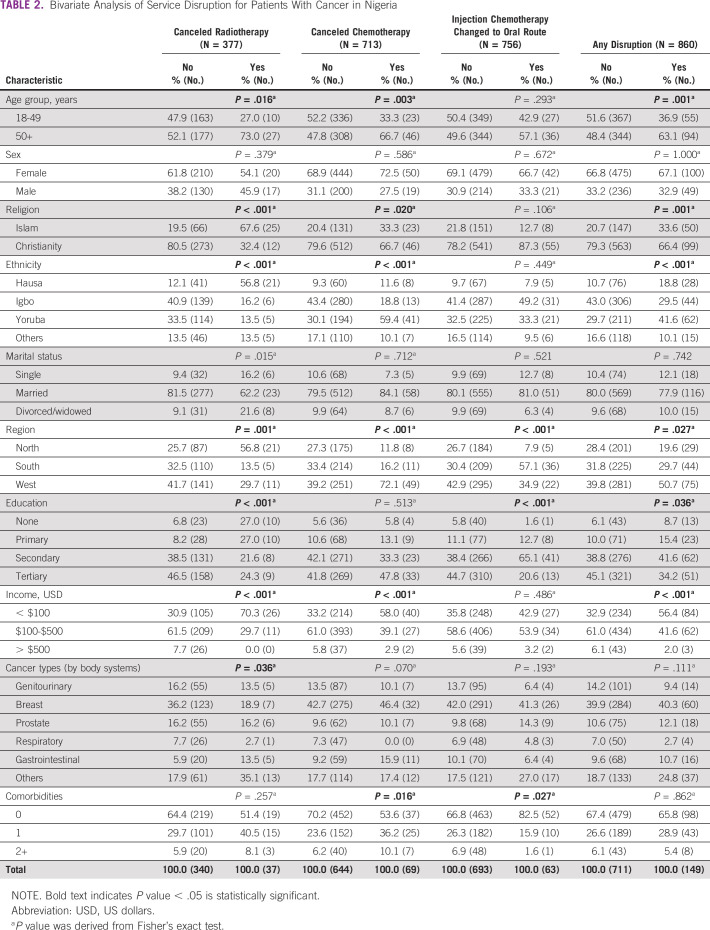
Bivariate Analysis of Service Disruption for Patients With Cancer in Nigeria

A similar pattern existed for radiotherapy cancellations. Older respondents age ≥ 50 years (*P* = .016), of the Islamic faith (*P* < .001), Hausa ethnicity (*P* < .001), married (*P* = .015), living in the Northern region (*P* < .001), with minimal education (*P* < .001), and an annual household income < $100 USD (*P* < .001) experienced increased rates of canceled radiotherapy appointments.

Similar results were noted for respondents reporting canceled chemotherapy appointments in terms of age (*P* = .003), religion (*P* = .020), and annual household income (*P* < .001), but they differed by ethnicity and region. There also appeared to be an association between individuals reporting having their chemotherapy canceled and low levels of perceived service provision (*P* < .001).

Modifications to chemotherapy regimens, specifically changing chemotherapy from injection to an oral route of administration, occurred more often among respondents who lived in the Southern region (*P* < .001), had secondary education (*P* < .001), or who had no comorbidities (*P* = .027).

### Multivariate Analysis of Service Cancellation

**FIG 2:**
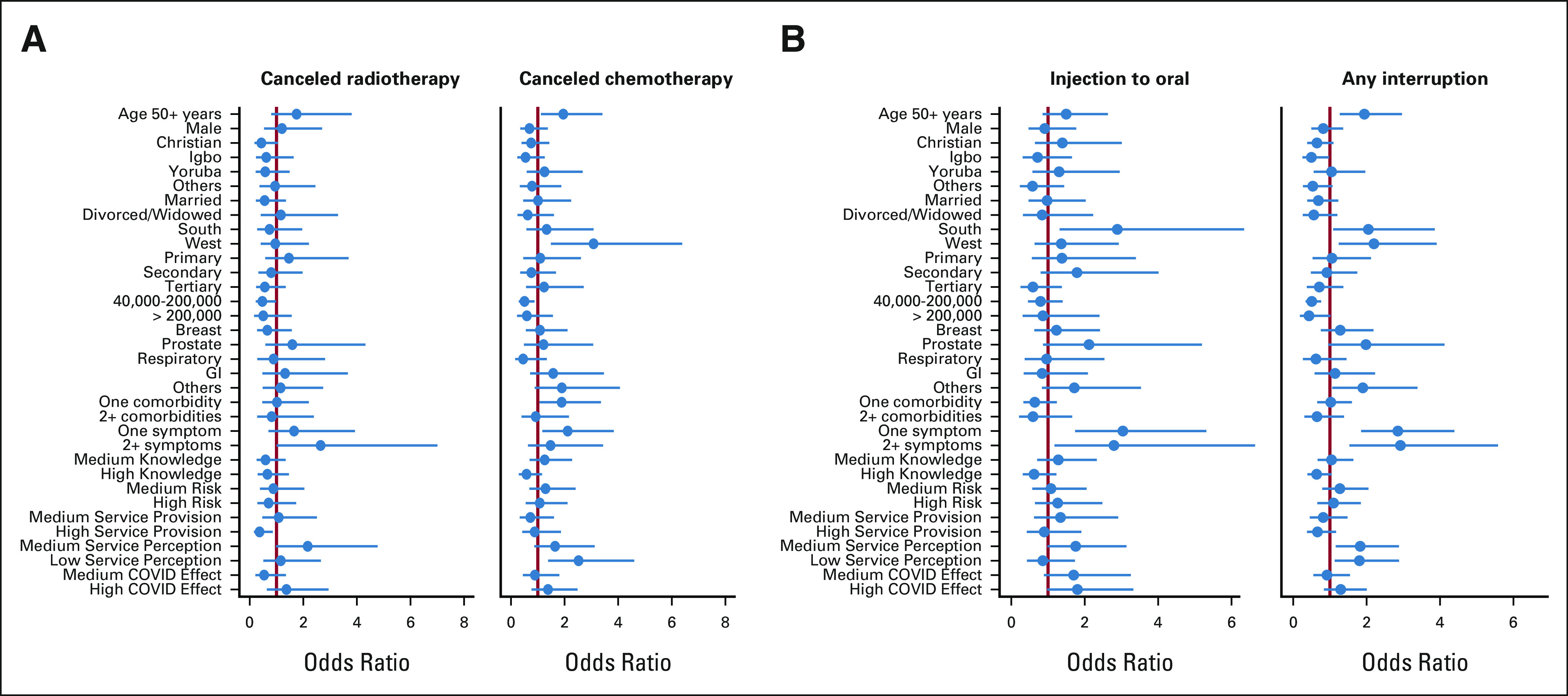
Multivariable analysis results.

Figure 2 displays multivariate analysis results of service disruptions and cancellations because of COVID-19 for Nigerian patients with cancer (Table 3). A detailed supplementary table with odds ratios and confidence intervals is presented (Appendix Table A1). The odds of experiencing a radiotherapy cancellation were highest for patients with prostate cancer, older patients, patients with a medium service perception, and those reporting two or more flu-like symptoms. For chemotherapy cancellation, odds of an appointment being canceled were higher for older patients, residents of the Western region, and those with low service perception (Fig 2A).

**TABLE 3:**
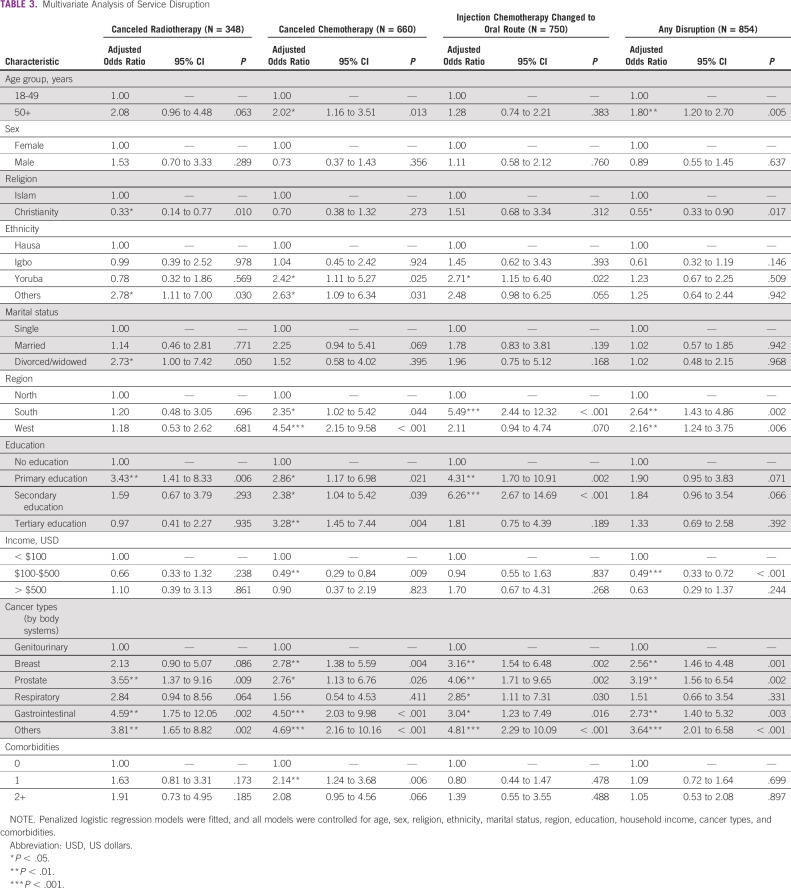
Multivariate Analysis of Service Disruption

The odds for changing chemotherapy route from injection to oral were highest for residents of the South region, patients with prostate cancer, those with one or more comorbidities (symptoms), and those experiencing high COVID effect (Fig 2B). Overall, odds for experiencing any disruption were highest for older patients, residents of the West, patients with prostate cancer, those with comorbidities/symptoms, and those with low/medium service perception.

## DISCUSSION

The COVID-19 pandemic has been described as one of the greatest challenges faced in recent years because of the severe impact on social, economic, and health care systems worldwide.^4^ Efforts to mitigate the spread of this virus has been a top priority for public health experts and professionals, with measures such as social distancing, improved personal hygiene, frequent handwashing with soap and water, use of alcohol-based hand sanitizers, and avoidance of crowded areas. Countrywide measures such as cancellation of air travel, closure of borders, enforcement of lockdowns of cities and states, and cancellation of social gatherings have been imposed in a bid to prevent the spread of COVID-19. The Nigerian Government, like other governments worldwide, instituted lockdowns in major cities as a means to reduce transmission of the virus in May 2020.^19^ As of December 2020, a total of 62,170 cases were recorded in the 12 states covered in this study or approximately 73.65% of the reported total case load for Nigeria.^8^

The impact on health care extended to the oncology centers with cancellation or postponements of treatments and the move to remote care, all in an effort to reduce spread and protect the patients and the health care professionals. Early studies had initially reported a possible higher risk of infection in patients on treatment for cancer, and many organizations released guidelines to direct changes to health care structures that would prevent transmission of the virus while trying to maintain care delivery to patients.^20^ Physical clinical visits were canceled, postponed, or changed to remote sessions with the use of virtual interaction technology; frequency of visits were reduced; many conventional radiotherapy regimens were altered to hypofractionated regimens; parenteral chemotherapy, where possible, was changed to oral; and nonemergent and elective procedures were postponed.^21,22^ Striving to find a balance between delivering optimal cancer care and mitigating risk of infection spread for patients and health care workers became a priority for oncologists and public health experts, with varying recommendations from within and outside the country.^23,24^

Nigeria, a resource-constrained, lower middle-income country has long struggled with limited oncology centers and facilities, inadequate infrastructure, and insufficient expert oncology manpower, all of which have resulted in documented gaps in cancer care even before the COVID-19 era.^25,26^ There are 54 health care centers in Nigeria providing tertiary-level health care services, with nine of them being classified as comprehensive cancer centers. Of these, eight centers had radiotherapy machines at the time of this study.^27^ As a result, patients with cancer often have to make interstate journeys to access expert and comprehensive oncology services.

This study evaluated patient-reported disruptions to cancer care during the first wave of the COVID-19 pandemic in Nigeria. Patient-reported outcomes have been documented to be more reflective of health status than clinical reporting, aiding clinicians in identifying patient needs and instituting appropriate interventions to improve patient satisfaction with care.^28^ We surveyed 1,079 patients with nationwide across 15 centers in the country to determine the impact the pandemic and the accompanying mitigation strategies had on access to care and cancer services across the three major geopolitical zones in Nigeria—North, South, and West. Patients from Northern, Southern, and Western regions accounted for 23.9%, 36.6%, and 39.5% of the study population, respectively. The high proportion of patients from the Western region, including states such as Lagos State, can be attributed to the presence of a comprehensive cancer center within the state that serves as a referral center for patients within and outside Nigeria. In the Northern region, Abuja state has the only functioning radiotherapy machines, and the lockdown instituted in the state in April 2020 is likely the reason for the lower proportion of patients seen in that region. The authors believe assessing this impact from a patient perspective would be valuable to drive improvements in cancer care delivery in the pandemic era.^29^

There was a preponderance of female respondents (65.7%), with breast cancer being the most prevalent cancer type recorded among respondents (37.3%). This is in line with the reported incidence of cancer in Nigeria, where women accounted for 66%-66.4% of patients with cancer in the population-based cancer registries covering a 2-year period from 2009 to 2010.^30^ Comorbidities were noted in 30.2% of respondents, similar to a 2018 study by Salako et al^31^ showing a 26.9% prevalence of comorbidities among surveyed patients with cancer in Nigeria.

Regarding the symptoms of COVID-19 infection in participants, the majority of participants reported none or only one symptom of the COVID-19 infection as at the time of review. This can be assumed to be reasonable as patients who would have possible symptoms of COVID-19 would have been discouraged from going out or would have been referred immediately to testing and isolation or treatment centers.

Many respondents scored high in COVID-19–related knowledge, likely attributable to majority having at least a secondary level of education. Approximately one fourth of the study respondents (24.0%) perceived their COVID-19 risk to be low, whereas one third of the patients (30.7%) perceived themselves to be at high risk of contracting COVID-19 because of their cancer diagnosis or treatment. Over half of the surveyed respondents (51.0%) reported medium to high levels of negative impacts of COVID-19 on cancer care. This suggests poor patient satisfaction, which can impair physical health- and mental health-related quality of life, reduce treatment compliance, and decrease motivation to seek care, all of which can result in poorer outcomes.^32^

Service disruptions in cancer treatment have previously been documented in Nigeria. In a recent survey of patients with cancer in Nigeria, 43% of respondents reported radiotherapy machine failures. Other reported causes of service disruption include treatment abandonment because of financial constraints, side effects, unavailability of medication, and loss to follow-up for unknown reasons.^33^

In this study, sociodemographic characteristics of participants were explored as possible social determinants of health and outcomes. This has been corroborated by other studies performed globally that have reported an association between social factors such as education, income, employment, marital status, and poor health status in patients.^34-36^ In addition, factors such as marital status are considered crucial, since they have been reported to contribute significantly to patient delay and, by extension, may result in poor clinical outcomes.^37,38^ In the current study, however, there was no statistically significant association between the marital status of participants and reports of any disruptions in care; although when canceled radiotherapy was taken singly, marital status was found to be associated.

Disruption experiences of participants in this study were consistent with a similar study carried out in Egypt by Abolkasem et al,^22^ where changes from parenteral to oral chemotherapy were recorded in 83.3% of surveyed centers. Disruption to treatment modality was significantly higher among patients age 50 years and above, compared with those age 18-49 years (*P* = .001). This is may be because older patients are at a higher risk of contracting the virus and are more likely to have comorbidities. Thus, health care workers are more wary of exposing this cohort of people to settings where they might contract the virus and are more likely to offer them alternate treatment options. Disruptions to treatment modality was also significantly higher in the West region, compared with other regions (*P* = .027).

Patients with a higher level of education had significantly higher levels of service disruption, compared with those with lower levels of education (*P* = .039). This was unexpected as people with lower levels of education were presumably more vulnerable and, therefore, were less likely to have access to services such as telemedicine, which was prevalent during the lockdown. However, perhaps this finding was due to the fact that more educated people were more likely to be more knowledgeable on COVID-19 preventive practices, thereby opting for virtual consultations and agreeing/requesting a change in regimen, compared with less educated individuals, which in turn was reported as a disruption to care.^39^ Similarly, respondents with higher levels of COVID-related knowledge experienced higher levels of service disruptions, compared with those with low levels of COVID-related knowledge (*P* = .018). This is also likely because of those having higher levels of knowledge being more likely to self-isolate in fear of contracting the virus, self-cancel their appointments, or opt for alternative treatment options to reduce the risk of infection.

People earning < $100 USD monthly had significantly higher levels of disruption to treatment, compared with people earning more (*P* < .001). This is likely because of the fact that patients with low socioeconomic status were more likely to suffer financial constraints and significant disruptions to livelihood as a result of economic restrictions during the lockdown. In a country where cancer treatment costs are almost exclusively out of pocket and 81.37% of the population are entrepreneurs or self-employed, shutdown of businesses and travel restrictions because of lockdowns would have a significant impact on those in the lower income bracket.^40,41^

Contrary to what might be expected, respondents with possible COVID-19 symptoms experienced significantly less service disruption to their treatments when compared with those without symptoms (*P* < .001). Varying trends in service disruption across the country may be an indicator of prioritization of oncology services in these regions, as well as education, income, and knowledge levels in the region. After multivariate logistic regression, significant predictors of service disruptions noted include age ≥ 50 years, Igbo ethnicity, divorced/widowed status, and patients from the South and West.

According to the National Institute for Health and Care Excellence rapid guidelines, avoiding radiotherapy if evidence suggests little or no benefit, deferring treatment if clinically appropriate, and shortening radiotherapy when possible were necessary to mitigate the spread of COVID-19. Changes from parenteral to oral chemotherapy agents or route of administration are also consistent with recommendations made by Nigerian oncology and public health experts.^42^ Adoption of other practices, including the use of hypofractionated radiation therapy, alterations in chemotherapy regimens, increased adoption of telemedicine, COVID-19 vaccination, prioritization of the care of patients with cancer, and case-by-case prioritization, may all help to maintain the standard of care for patients with cancer, even considering the limitations of the pandemic.^21,43^

Although the current study did not cover the scope of the implications of these service disruptions on clinical outcomes, previous studies have reported increased mortality in cancer patients' consequence of delays or disruptions in care. Gupta et al^44^ in India documented an estimated increase in cancer-associated mortality of 2.52%-3.80% owing to treatment interruptions because of COVID-19 in patients with cervical cancer. In Nigeria, where patients already typically present with advanced stages of cancer, we postulate that the increase in cancer-associated mortality may be similar, if not more, than was recorded in India. However, additional follow-up studies are necessary to determine the impact of COVID-19–related disruptions to care on the cancer-associated mortality rate during the pandemic.^45^

This study has potential limitations. The study relied on patient-reported disruptions to care and may be subject to bias. The inclusion of centers representative of all the geopolitical zones in the country and large sample size was one of the ways to try to mitigate this potential bias. In addition, it can be argued that the patients who experience disruptions because of COVID-19 symptoms may have been missed, since they were asked to remain at home and self-isolate and, as such, may have been unavailable during recruitment to review for disruption. However, since oncology treatment occurs over a long period of time and data collection for this took place over a period of 4 months, this would ensure that these patients were surveyed after the resolution of their acute illness because of COVID-19, and they would have visited the center at least once during the study period. Finally, the cross-sectional nature of the study design makes it difficult to determine causality; however, the study noted useful significant associations between the factors potentially contributing to care disruptions in patients with cancer in Nigeria during the COVID-19 pandemic.

The COVID-19 pandemic resulted in major disruptions to social, economic, and health care systems all around the world. In this Nigerian study, we found that one in 10 patients experienced disruptions in cancer care, and one in two experienced social or economic disruptions to accessing care, with disruptions most prominent in individuals with more constrained resources. As such, policies and strategies aimed at minimizing service disruptions while maintaining safety for patients with cancer are necessary. Strategies such as the implementation of digital technology to improve health care access, adaptation and adoption of treatment regimens that require fewer physical hospital visits such as hypofractionated radiation therapy regimens, and oral chemotherapy drugs, and regimens such as capecitabine-based treatment in place of fluorouracil should be a priority for all Nigerian cancer care institutions in the COVID-19 era. Furthermore, nationwide implementation of COVID-19 vaccination, prioritization of the care of people with noncommunicable diseases such as cancer, and case-by-case prioritization may all help to maintain the standard of care for people living with cancer, even considering the limitations of the pandemic.

## Figures and Tables

**TABLE A1:**
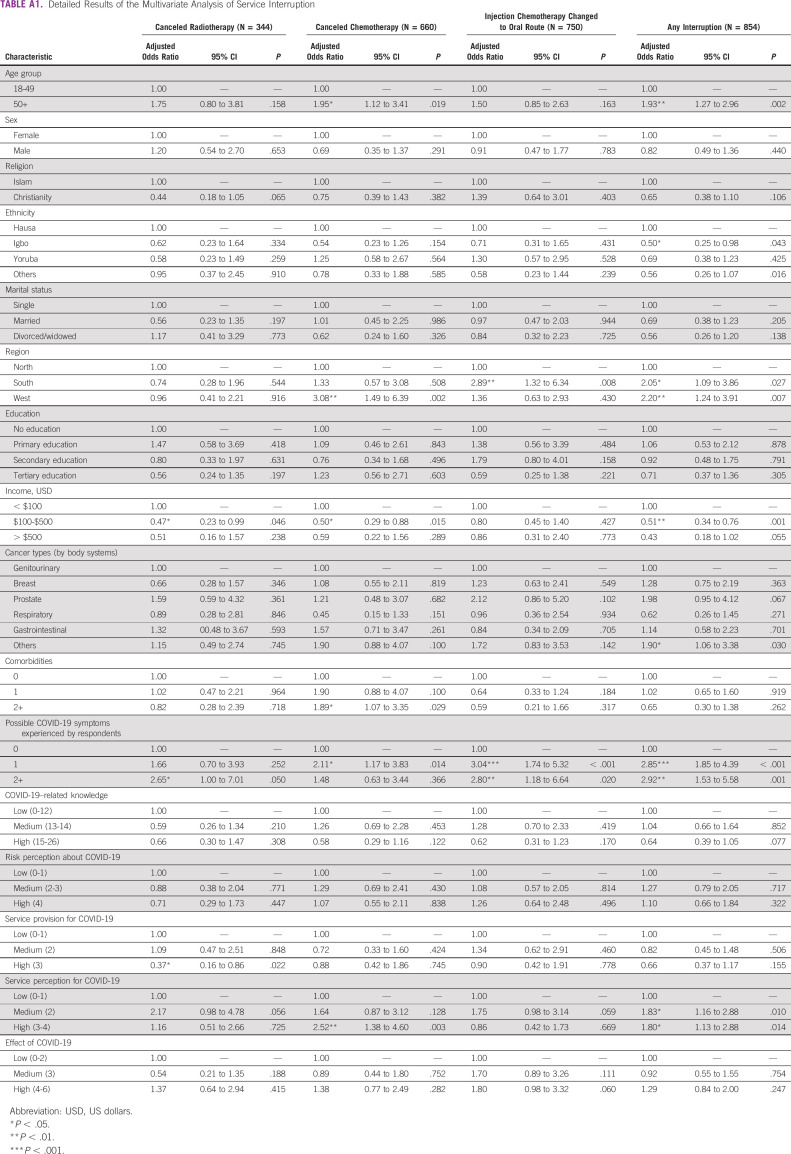
Detailed Results of the Multivariate Analysis of Service Interruption
